# Clinician Experiences With Hybrid Closed Loop Insulin Delivery Systems in Veterans With Type 1 Diabetes: Qualitative Study

**DOI:** 10.2196/45241

**Published:** 2023-03-29

**Authors:** Kara Mizokami-Stout, Holly M Thompson, Kathryn Hurren, Virginia Leone, Gretchen A Piatt, Joyce M Lee, Rodica Pop-Busui, Melissa DeJonckheere

**Affiliations:** 1 Division of Metabolism, Endocrinology and Diabetes Department of Internal Medicine University of Michigan Ann Arbor, MI United States; 2 Ann Arbor Veterans Affairs Healthcare System Ann Arbor, MI United States; 3 Department of Learning Health Sciences University of Michigan Ann Arbor, MI United States; 4 Division of Pediatric Endocrinology Department of Pediatrics University of Michigan Ann Arbor, MI United States; 5 Department of Family Medicine University of Michigan Ann Arbor, MI United States

**Keywords:** automated insulin delivery, hybrid closed loop, type 1 diabetes, endocrinology, qualitative study, insulin delivery, digital health intervention, thematic analysis, diabetes management

## Abstract

**Background:**

Hybrid closed loop (HCL) insulin pumps adjust insulin delivery based on input from a continuous glucose monitor. Several systems are FDA approved and associated with improved time in range, reduction in hemoglobin A_1c_, and decreased incidence of hypoglycemia. Major diabetes guidelines differ in their strength of recommendations regarding the use of HCL systems. Overall, limited information about the factors that influence HCL pump clinical decision-making is available, especially among endocrinology clinicians.

**Objective:**

The study objective is to describe the knowledge and attitudes, network support, and self-efficacy regarding HCL insulin delivery systems among endocrinology clinicians in one Veterans Affairs (VA) Healthcare System in the Midwest.

**Methods:**

Following a descriptive approach, this qualitative study used semistructured interviews and inductive thematic analysis. All endocrinologists, endocrinology fellows, and nurses in the endocrinology and metabolism department at one VA Healthcare System in the Midwest were invited to participate in one-on-one phone interviews. Thematic analysis explored clinician perspectives on HCL insulin pump systems.

**Results:**

Participants (n=11) had experience within VA and university health care system endocrinology clinics. From their experiences, 4 themes were identified involving the evaluation and assessment of insulin pump candidates, prescribing challenges, clinical benefits of HCL pumps, and overall clinician confidence.

**Conclusions:**

Findings suggest that clinicians believe HCL systems have significant glycemic benefits but are not appropriate for all patients, especially those with cognitive impairment. HCL pump initiation is a multi-step process requiring an interdisciplinary team of health care clinicians to ensure patient and pump success. Furthermore, HCL systems improve clinician confidence in overall diabetes management.

## Introduction

In 2016, the first commercial “artificial pancreas” or hybrid closed loop (HCL) insulin delivery system was made available [1]. HCL systems use a continuous glucose monitor (CGM) and control algorithm to automatically adjust insulin delivery based on current and predicted sensor glucose values [2]. HCL systems can either adjust basal insulin delivery, provide small correction boluses, or both; however, mealtime boluses are not automated and rely on the patient to count and input carbohydrate data. Currently, 3 HCL systems are approved by the US Food and Drug Administration (FDA). The MiniMed 670G with Guardian 3 CGM system (Medtronic PLC) was the first HCL system, becoming available in September 2016. The t:slim X2 pump with Dexcom G6 CGM (Tandem Diabetes Care, Inc) has been available since December 2019. The Omnipod 5 with Dexcom G6 CGM (Insulet Corp) was approved by the FDA in January 2022.

Several studies have reported many glycemic benefits to the use of HCL systems [3,4]. These studies demonstrate that HCL systems increase time-in-range (TIR) up to 10% overall and 15% overnight compared to older insulin pump technology. Data also suggests an additional reduction in hemoglobin A_1c_ (HbA_1c_) by about 0.5% with an HCL pump versus regular or sensor-augmented insulin pumps alone. Use of an HCL system for 6 months was associated with an approximately 1% decrease in time spent in clinically significant hypoglycemia (ie, level 2 hypoglycemia, defined as a blood sugar level below 54 mg/dL). The improvements in HbA_1c_ and TIR are presumed to translate to a reduction in microvascular complications [5,6].

Due to these clinical advantages to HCL pump use, diabetes clinical practice guidelines routinely recommend the use of HCL systems for both adults and youth with type 1 diabetes. The American Diabetes Association (ADA) guidelines state automated insulin delivery systems of HCL pump systems may be offered for diabetes management in all adults and youth with type 1 diabetes (grade A: clear evidence from randomized trials) and other types of insulin-deficient diabetes (grade E: expert consensus) [7]. Similarly, the recently published American Association of Clinical Endocrinology (AACE) guidelines recommend automated insulin delivery systems for “many” patients with type 1 diabetes (grade A: high strength of evidence; best evidence level) [8]. As both guidelines emphasize, there are a variety of insulin delivery modalities, and the use of HCL systems depends on a variety of factors, including patient preference, caregiver preference (if applicable), provider preference, patient and clinic resources, and payer considerations [7,8]. Recent studies demonstrating racial and ethnic disparities in diabetes technology for type 1 diabetes highlight the importance of understanding why discrepancies in the use of HCL systems exist [9].

**Figure 1:**
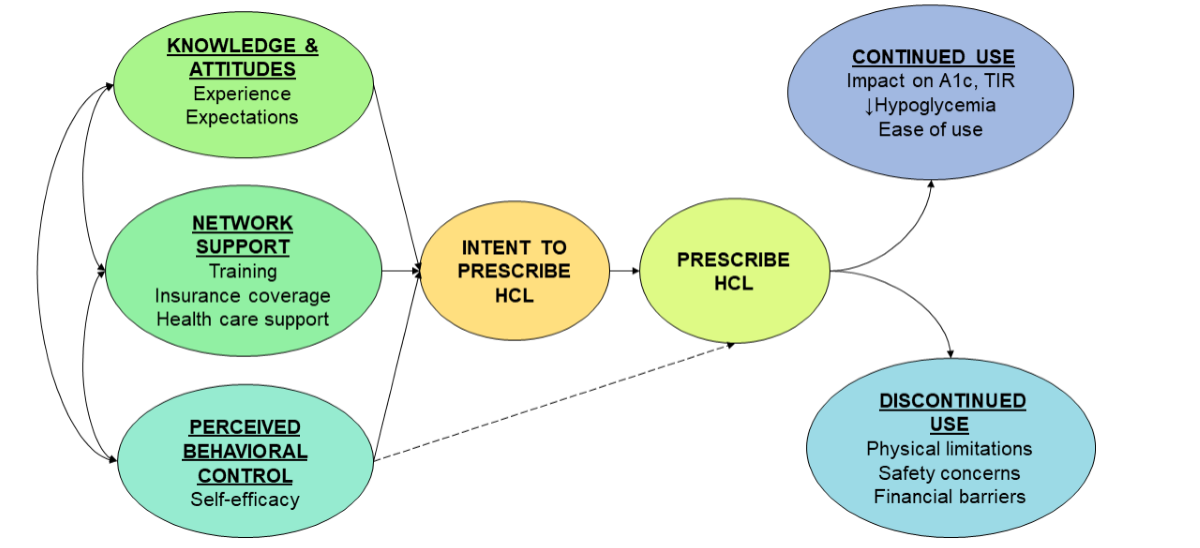
Conceptual model based on modified Theory of Planned Behavior [14]. HCL: hybrid closed loop; A_1c_: hemoglobin A_1c_; TIR: time-in-range.

Although endocrinologists are experts in diabetes care and use of diabetes technology, there is limited literature available regarding the factors that influence their decisions regarding HCL systems in type 1 diabetes. Previous qualitative studies regarding diabetes technology examined the perspectives of certified diabetes care and education specialists, research nurses, diabetes nurse specialists, and dieticians; very few included information from endocrinologists [10-12]. These studies also lacked rigorous methodology in that they were not rooted in known behavioral theory, or they used survey data alone, which lack the richness and complexity of semistructured interviews, considered the gold standard for qualitative research [13]. Additionally, data on HCL systems is especially lacking in veteran populations with type 1 diabetes. To address these gaps, this study aims to describe the knowledge and attitudes, network support, and self-efficacy regarding HCL insulin pump systems among endocrinology clinicians at one Veterans Affairs (VA) Healthcare System in the Midwest based on the modified Theory of Planned Behavior, as shown in Figure 1 [14].

## Methods

### Ethics Approval

This study was approved by the VA Ann Arbor Institutional Review Board and Research and Development Committees (1618650).

### Data Collection

Semistructured interview guides were developed to assess the knowledge and attitudes, network support, and self-efficacy of endocrinologists based on the Theory of Planned Behavior [14] (Multimedia Appendix 1). All the endocrinology physicians and nurses (N=13) from one VA Healthcare System in the Midwest were invited to participate in the interviews via email. Upon expressing interest in participating, they were provided with a copy of the study cover letter and informed consent document. One author (HMT) conducted interviews by phone from December 2021 through February 2022. Although the interviewer followed the interview guide, semistructured interviews are adaptable and allow for a loose, flexible structure to aid in discussion and insight into participant perspectives. This facilitates a deeper exploration of participant thoughts and experiences while gathering detailed information [13]. The interviewer completed a reflection form immediately following each interview that included the main ideas and initial thoughts on the interview process. Interviews were audio recorded with 2 types of recording system, Audacity (Audacity Team) and the Olympus 9500 DVR (Olympus Corp), in case of technical failure. Immediately after each interview was completed, the recording was uploaded to a secure server, where it was then deidentified and transcribed verbatim into Microsoft Word (Microsoft Corp) to prepare for data analysis by the research team.

### Data Analysis

**Figure 2:**
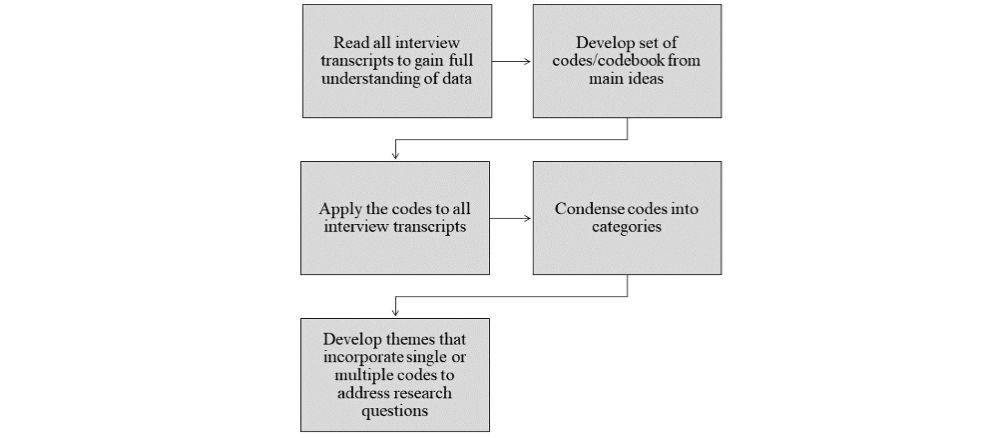
Inductive thematic analysis process.

As described in Figure 2, thematic analysis was used for the interview transcripts. NVivo (version 12; QSR International) was used for data management and organization. We inductively developed a coding template that reflected participant responses during the interview (Multimedia Appendix 2), which was initially used to code each transcript, though codes were iteratively modified and updated throughout the coding process to ensure all concepts were represented. Codes were further condensed into themes and participant quotes were used to support identified themes. One author (HMT) initiated coding of all transcripts and organization of themes. Themes were developed and discussed in detail with all authors to reach agreement and conclusions. Data analysis was completed as an ongoing process as the scheduled interviews were completed with each provider. Throughout the process the research team documented aspects of the qualitative study design using the COREQ (Consolidated Criteria for Reporting Qualitative Research) guidelines (Multimedia Appendix 3) [15].

## Results

### Overview

Interviews were conducted at one VA Healthcare System in the Midwest among endocrinology and metabolism clinic staff, the majority of whom also had a dual appointment at the University of Michigan. Of the 13 approached physicians and nurses, 11 (for a response rate of 85%) agreed to participate in the interviews. Clinician characteristics are displayed in Table 1. Interviews lasted a mean 37 (SD 9.15) minutes.

Overall, participants provided insight into their experiences at both the Midwestern VA Healthcare System and the affiliated university health care system. From the interviews, 4 themes were identified to encompass the knowledge and attitudes, network support, and self-efficacy of clinicians regarding the use of HCL systems, as shown in Textbox 1.

**Table 1:** Participant characteristics.

Characteristics			Participants (n=11)
**Gender, n (%)**			
	Male	5 (45)	
	Female	6 (55)	
**Clinician type, n (%)**			
	Endocrinologist	4 (36)	
	Endocrinology fellow	5 (46)	
	Diabetes nurse	2 (18)	
**Practice site, n (%)**			
	Veterans Affairs health system only	2 (18)	
	Veterans Affairs and university health system	9 (82)	
Clinical experience (years), mean (SD)			16.5 (11.80)
Clinician confidence rating in diabetes technology (scored from 1 to 10), mean (SD)			7.9 (1.13)

**Summary of themes for endocrinology perspectives on hybrid closed loop systems in type 1 diabetes.**

**Ongoing hybrid closed loop (HCL) candidacy**

Initial and continued assessment for HCL appropriateness is important for optimal use.

**HCL initiation barriers**

Challenges persist in initial procurement, education, and training, despite insurance coverage.

**HCL benefits in real-world use**

Clinical benefits include improved glycemic control and reduced hypoglycemia.

**Improved clinician confidence**

Enhanced clinical decision-making and overall confidence in providing care for patients with type 1 diabetes.

### Ongoing HCL Candidacy

Clinicians felt strongly that HCL systems are not a one-size-fits-all approach to diabetes care. Instead, patients should be carefully considered for these systems to ensure overall appropriateness and optimal clinical benefit. Participants felt that patients who are comfortable with technology and are highly motivated to manage their diabetes make suitable candidates for HCL technology. Additionally, several clinicians reported that the HCL systems are most beneficial for patients with variability in their day-to-day schedules, especially regarding meals.

I think patients who have a lot of variability in their schedule, and whether that sort of work, sleep schedule, or whether that’s like, when do they exercise, when are they active, I think those patients are a group that really benefits.

Endocrinologist 3

Clinicians also felt there are patients who are not ideal HCL-system candidates. Some of the most common examples included patients with limited dexterity, patients with impaired cognition, and patients who are not technology savvy, as they might be significantly more likely to be frustrated by complex technology. Clinicians also commonly brought up the example of patients who still struggle with carbohydrate counting and noted that this is a significant limitation to HCL pump use, since these systems still require patients to enter carbohydrate values to receive input for food boluses. Clinicians were surprised that patients who have very high expectations and demand perfect blood sugar control struggle with a system that provides so much real-time data. They described how these patients may become overly concerned about their blood glucose readings, leading to inadequate boluses of meal-time insulin and the inability to trust the HCL system when necessary.

Patients are often not used to seeing what their blood sugars look like at all times of day and my experience is that they tend to get nervous a little bit more.

Endocrinologist 3

Participants in our study described how each patient must be continuously assessed to ensure appropriate use, even if the clinicians believed they were an appropriate candidate. If at any point the clinician felt that the pump was potentially harmful, the patient was transitioned back to a multiple-daily-injection insulin regimen. Across participants interviewed, the most common reason for any previous pump discontinuation was development of cognitive decline or dementia, as summarized here:

Probably the most common reason we have to take people off a pump or take it away from them is dementia, altered mental status where they just become incapable of managing the pump anymore...

Nurse 1

One endocrinologist offered valuable insight into their streamlined approach for initial patient evaluation and assessment. By using a stepwise approach, the clinician slowly added on different components of the HCL system to ensure the patient felt comfortable with each aspect of the technology.

For somebody who is doing insulin injections but is [already] monitoring their glucose using a continuous glucose monitor, that may be an easier transition to a pump with a hybrid closed loop system because they already know one piece of the technology.

Endocrinologist 3

Overall, the provider assessment and evaluation of patients for HCL candidacy was highly individualized, reflecting their years of clinical experience and different examples of patients they had cared for during various clinic encounters.

### HCL Initiation Barriers

Clinicians commonly commented on the frequent challenges they experienced when first prescribing an HCL system. Most clinicians interviewed had experience at the VA and university health system and could easily discuss key differences between the 2 practice sites. For example, clinicians described the differences in insurance coverage for patients in the 2 systems, noting the added complexities of describing HCL pumps in the university health system.

[One of the challenges is] whether this will be covered by a patient’s insurance or not and what their copay would be and then maybe the time that it takes for a patient to schedule all the appropriate appointments and learn about the pumps. So, it’s sort of time consuming.

Fellow 5

Surprisingly, clinicians conducting clinic visits directly with patients and monitoring or adjusting their insulin therapy were not the same clinicians who placed orders for HCL pumps within either health system. In both health systems, HCL ordering is more complex than a typical prescription, and although a physician signature is required, the actual order process is completed by clinic support staff. At the VA, the nurse manager for the endocrinology clinic typically places all insulin pump orders to ensure a consistent process. In the university health system, medical assistants typically placed the orders for the insulin pump and supplies, but clinicians described the specific and detailed documentation that was required from their end for HCL pump approval.

At the University we go through our diabetes educators, and they will do the insurance authorization, the teaching, and once they’ve done those things, they’ll send a script for us to sign, and they try to mimic kind of their basic setting based on a current regimen.... It’s similar at the VA via the pharmacist.

Fellow 1

Another frequent and key difference that was mentioned by clinicians was the difference in resources available between the VA and university clinics. Unique to this specific VA facility, the endocrinology service offers a clinic a half day per week that is dedicated to just HCL pump management. This clinic offers interdisciplinary health care professionals, including a clinical pharmacist, nurses, and an insulin pump company representative, in addition to the endocrinologists, to provide support, answer patient questions, and provide initial education and training to new insulin pump patients. In contrast, the university health system has a team comprising certified diabetes educators that deliver the initial pump education, training, and monitoring.

Additionally, clinicians discussed various challenges patients face while initiating use of an HCL insulin pump. Most of the difficulties originate from the acclimation process. In some instances, patients are completely inexperienced in using any continuous insulin-infusion devices. In other cases, patients have to adjust to the differences between the HCL system and their older pump technology. It is also challenging for patients to begin to trust the technology and adjust to the availability of monitoring blood glucose in real time via a CGM system that communicates directly with the insulin pump.

Trusting the technology, a lot of patients have had diabetes for a very long time and they’re just very used to controlling their own blood sugars and kind of giving up a lot of that control to a little machine, I think some patients do have a little difficulty with that, especially some of the older folks.

Endocrinologist 4

Other challenges included difficulties with obtaining and ordering pump supplies and the infusion sets and with sensors appropriately adhering to the patients’ skin. Determining initial insulin rates could also be difficult, especially if a patient was new to the clinic or health system in addition to being new to insulin pump technology.

To mitigate these challenges, clinicians often reported relying on assistance from interdisciplinary team members for support and troubleshooting of malfunctioning devices or technology glitches. Other nonclinic resources that were reported as beneficial to patients included YouTube videos and direct patient contact, usually via telephone or website, with the insulin pump company or manufacturer.

### HCL Benefits in Real-World Settings

Another theme that clinicians commonly discussed was the clinical benefits of HCL insulin pumps. In general, clinicians noticed improved glucose control with less variability and fluctuations in patient blood sugars. Importantly, clinicians noted the reduction in hypoglycemia, especially nocturnal hypoglycemia and hypoglycemia surrounding physical activity.

Much-improved nocturnal control with much fewer fluctuations especially less hypoglycemia or unexplained hypoglycemia at night and tends to be better postprandial, I see more control in most patients, so variability in it goes down; some moderate, modest to moderate improvement of overall A_1c_, and fewer hypoglycemia events.

Endocrinologist 2

I think the hypoglycemia incidence seems to be improved and just kind of like the yo-yo of going up and down or the variability of it just seems to be improved for patients.

Fellow 3

Many other clinicians interviewed also mentioned an overall improvement in hemoglobin after HCL initiation.

Besides the clinical improvements in glycemic control, HCL systems also have social benefits. Clinicians reported these insulin pumps allowed for greater convenience and flexibility for patients, especially in the timing and content of meals throughout the day.

The flexibility is...you’re not chained to having to eat a certain amount at a certain time. I think that’s the greatest thing that it does for the patients. It sort of allows them to you know have brunch on Sunday, they don’t have to [know] what time dinner is, they don’t have to worry about bottoming out because of their NPH [insulin] that they took earlier.

Endocrinologist 1

While overall attitudes toward HCL pumps were positive, clinicians also discussed several clinical and social limitations. Clinicians recognized the pump algorithm was not perfect and could sometimes lag when correcting patients’ hyperglycemia, especially postprandially. Other clinicians suggested it would be beneficial if there were more customizable features to the pumps, such as setting individualized glucose targets.

I would like to see in a system, at least for the physicians, to have more latitude in setting the target glucose. They largely have one main target glucose and then there’s sort of an activity type target you can set temporarily, but I would like to see it more customizable.

Endocrinologist 3

Note the recent FDA-approved Omnipod 5 system does include software with adjustable glucose targets; however, no clinicians that participated in interviews had patients on this system. Social limitations discussed consistently by the cohort of clinicians interviewed included challenges with pump alarms and device adhesion. Clinicians reported some patient dissatisfaction with being attached to a device for all hours of the day. Patients also reportedly struggled with sensors falling off or malfunctioning, thereby interrupting or diminishing their ability to use the HCL functionality of the pump. Other problems included the frequency of alarms with certain systems, as well as the number of calibrations with a glucometer that were required while using a particular HCL system. One provider commented on the importance of educating patients before they started a pump on the impact fingerstick blood glucose checks may still have on their lives.

I try to include that conversation that [CGM] is not a replacement of finger sticks and that it’s really just going to reduce the frequency that you’d have to finger stick, but I do think that the calibration of the [certain hybrid closed loop] systems is something that’s not as attractive for patients.

Fellow 3

### Improved Clinician Confidence

Clinicians interviewed had an average self-reported confidence rating in prescribing and managing diabetes technology of 8 (on a 1 to 10 scale, with 1 being the least confident to 10 being the most confident). However, upon further discussion of overall knowledge and competence in diabetes management, all clinicians reported HCL insulin systems added to their confidence. Clinicians highlighted that much of their confidence was due to these pumps often resulting in less hypoglycemia, a potentially dangerous side effect of poor insulin management, leading to a perception of safer care for patients.

You feel a little bit better that the pump is helping me and the patient when if maybe I haven’t programmed or had a chance to figure out their settings perfectly because I just don’t have enough time with them yet. You know when they’re new to me or I don’t have a lot of experience with their diabetes or when the patient’s just not doing everything exactly as they’re supposed to. So, I think it gives me some confidence that they’re less likely to get into trouble.

Endocrinologist 3

I think I feel more comfortable because I’m thinking that it will help prevent hypoglycemia, which is something scary in diabetes, so I think it’s keeping patients safer.

Fellow 4

Since these systems not only contain a CGM device, but also include the capability for that device to communicate with the insulin pump in real time, increased data are available to help make clinical decisions based on underlying physiology and patient behaviors. It is well-known that glucose trends available from CGM devices facilitate shared decision-making and goal setting in patients with diabetes [16]. However, HCL devices also augment insulin delivery in real time in response to glucose trends and provide information on patient bolusing behaviors. Clinicians attributed their increased confidence and competence in diabetes management to this increased amount of information, which could be used to identify patterns and guide clinical decision-making.

I feel like it’s definitely helped me understand patterns more and understand where we can make changes and how to make those changes. It kind of helps give more guidance with all the data that it provides too.... What’s nice is when you see when the basal rates change, especially if there’s an acute change, it kind of tells you, “oh that means more insulin is needed for their meals because their basal shot up all of a sudden,” or it’s really dropping down, “oh, that’s because they’re having tight sugars or low sugars at the point of the night or day.” So, seeing that also kind of tells you what’s actually going on underneath.

Fellow 2

In general, the attitudes of this cohort of endocrinology clinicians were positive toward the use of HCL insulin-delivery devices. Clinicians reported that they enjoyed working with the devices, despite their limitations, to continue to provide the best possible diabetes care to their patients.

## Discussion

### Principal Findings

This study sought to describe the knowledge and attitudes, network support, and self-efficacy of endocrinology clinicians regarding HCL insulin delivery systems within a VA health care system. Key themes identified included the evaluation and selection criteria used by professionals for HCL candidacy, prescribing challenges, perceived clinical benefits, and clinicians’ confidence in diabetes management. Although other qualitative analyses have detailed similar results in survey studies, this study is unique in the patient population (veterans with primarily adult-onset type 1 diabetes), provider population (specifically endocrinology physicians and nurses), and technologies assessed.

Our data support findings from Lawton et al [17] made after interviewing 12 diabetes nurses and 6 dieticians; they also perceived that, in general, insulin pumps offered better self-management to patients. However, the staff used a variety of clinical and patient-specific personal and psychological attributes as criteria to select patients for pumps, and this affected the staff’s perceptions of the benefits of HCL pump technology. In line with our findings, patients with unpredictable or highly physically active lifestyles and those who were technology savvy were more likely to be selected for HCL systems, while a history of poor adherence to self-monitoring or medications or an expectation that the pump would take over all aspects of diabetes management predicted unsuccessful outcomes with an HCL system. This is concerning, as studies have demonstrated the utility of HCL systems for individuals across the HbA_1c_ and control spectrum. This could lead to inadvertent differential access to technology, which in turn could affect clinical outcomes. Efforts are needed to ensure equitable access to HCL systems regardless of HbA_1c_ or patient characteristics [9].

We also found that access to technology in general was an important resource for HCL use, which is similar to findings from an Australian study of semistructured telephone interviews that identified themes related to access to HCL system technology and available support [12]. Limited qualitative data are available about HCL insulin pumps, especially regarding clinician attitudes and experiences with their use. In contrast to previous studies, interviews from this study provide insight on a wide range of attitudes and experiences, including clinical benefits, prescribing trends, HCL candidacy, and overall provider confidence in diabetes technology.

Consistent with our findings, a study that included multidisciplinary health care professionals in the United Kingdom found that financial resources and insurance coverage were critical for HCL device use [18]. Importantly, these prior studies were conducted in countries with significantly different health care systems and access to care. This study found that clinicians practicing within a United States VA health care system perceived that HCL system access for appropriate patients was better than at a major university hospital system, likely due to the elimination of insurance barriers.

Additionally, clinicians interviewed in our study frequently discussed the multiple benefits of these systems, such as improved glucose control, reduced glucose variability, and a lower incidence of hypoglycemia. These results mirror the clinical benefits seen in HCL clinical trial data [3,4]. Each provider had individual screening mechanisms, in addition to predefined VA criteria for use, when determining if a patient would make an adequate pump candidate.

### Strengths and Limitations

This study had several strengths. The first is that a theory-based assessment was used, which adds to the overall rigor of the study. This study is also novel in that the interviewees were all endocrinology clinicians, the majority of whom practice in both academic and VA heath care systems, which adds to the generalizability of our findings. While only 11 clinicians were interviewed, they represented 85% of all the eligible endocrinologists (ie, all but 2 clinicians invited elected to participate). Additionally, clinicians had experience with all HCL insulin pump systems that were available to patients at the time the study was conducted.

Limitations include that the study took place at a single-center VA health care system. Other VA health systems or endocrinology practices may have different processes, use different HCL systems, or have alternative clinical personnel to execute tasks such as insulin pump starts. Another limitation is that few data were gathered and analyzed regarding the familiarity of the clinicians with each HCL system used, their preferences for specific HCL systems, or differences in roles and training in each clinician group: endocrinologists, endocrinology fellows, and nurses. However, the purpose of this study was not to look for explicit differences, but instead look for similarities in experiences of the various clinicians on a care team. Future research could further examine whether there are differences between groups, which would require access to a larger sample of diabetes care team members.

### Conclusion

This qualitative analysis of semistructured interviews provides insight into the knowledge and experiences of clinicians practicing in an endocrinology clinic in a VA health care system. Our study is the first to include US VA clinicians such as endocrinologists and fellows that are prescribing and monitoring patients on these devices. Our findings suggest that HCL systems have significant glycemic benefits but are not appropriate for all patients, especially those with dementia or other degrees of cognitive impairment. HCL pump initiation is a multistep process requiring an interdisciplinary team of health care clinicians to ensure patient and pump success. Furthermore, HCL systems improve clinician confidence, knowledge, and competence in diabetes management. Additional studies are needed describing non–endocrinology provider knowledge and attitudes, as well as patient experiences, to provide a complete assessment of HCL system access and impact on diabetes care and quality of life.

## Appendix

Multimedia Appendix 1 Endocrinology clinician semistructured interview script.

Multimedia Appendix 2 Codebook based on the modified Theory of Planned Behavior.

Multimedia Appendix 3 Consolidated Criteria for Reporting Qualitative Research (COREQ): a 32-item checklist for interviews and focus groups.

## Abbreviations

AACE: American Association of Clinical Endocrinology
ADA: American Diabetes Association
CGM: continuous glucose monitor
COREQ: Consolidated Criteria for Reporting Qualitative Research
FDA: US Food and Drug Administration
HbA_1c_: Hemoglobin A_1c_
HCL: hybrid closed loop
TIR: time-in-range
VA: Veterans Affairs Healthcare System
