# Supplementary material for: Clinician Experiences With Hybrid Closed Loop Insulin Delivery Systems in Veterans With Type 1 Diabetes: Qualitative Study
Source: JMIR Diabetes. 2023 Mar 29;8:e45241. doi: 10.2196/45241 (PMC10132000; doi:10.2196/45241)
Supplement: Multimedia Appendix 1 [file diabetes_v8i1e45241_app1.docx]

**Appendix 1**

Endocrinology clinician semi-structured interview script.

**Script:**

Welcome. I would like to thank you in advance for your participation today.

**(Purpose)**

The purpose of this interview is to better understand your experience with hybrid closed loop or “artificial pancreas” insulin delivery systems, specifically the Medtronic 670G system and the Tandem T-slim/Dexcom with Control IQ systems. I have these questions as a guiding post but I would like this to be a very fluid conversation.

**(Human Subjects Projection Procedure)**

You should know that your responses to these questions are private and we will not share any identifiable information.

**(Icebreaker)**

To start, do you provide care for patients with diabetes in your clinic? If so, do you see patients with type 1 diabetes, type 2 diabetes or both? If you see type 1 diabetes, what percentage of your overall patients have type 1 diabetes?

**(Opening question)**

Can you tell me about your experiences with diabetes technology?

**Domain 1: Knowledge and attitudes**

**(Example questions)**

- How do you learn about new diabetes technology?
- What is your experience with the currently available HCL systems?
- What benefits do these systems have for your patients? Are there populations that you feel benefit more than others?  Are there populations that you feel may not be appropriate for these systems?

**If not mentioned, ask more specifically about type 1 versus type 2 populations*

- How have HCL systems met your expectations?
- How have HCL systems failed to meet your expectations?

**Topic to explore: Network support**

**(Example questions)**

- What is the process of prescribing an HCL system look like in your practice?
- What is the process of managing and HCL system?
- Probing:

-What challenges do you face in initiating patients on these systems?

- What does the process for following up patients on these devices look like?
- Probing questions:

-What clinic support is in place to access HCL data?

-What clinic resources do patients have access to?

-What clinic support is in place to help troubleshoot technology difficulties?

- What are the most important challenges that patients face as they start on HCL systems?
- What is your protocol to support patients who are struggling on HCL systems?

**Topic to explore: Perceived behavioral control**

**(Example questions)**

- How confident (from 1-10) are you in prescribing and managing new diabetes technology?
- Have HCL systems helped or hindered your confidence in diabetes management?

**(Closing opportunity for spontaneous information)**

- Is there anything I didn’t ask that you feel I should know?

**(Follow-up planned)**

Our goal with this interview is to better understand the experiences of providers who providers who care for patients with diabetes who may be eligible for HCL systems. Ultimately, our goal is to understand how we can better improve both patient and provider experiences with these devices in the future. Thank you so much for your participation in this interview.
