# Supplementary material for: Clinician Experiences With Hybrid Closed Loop Insulin Delivery Systems in Veterans With Type 1 Diabetes: Qualitative Study
Source: JMIR Diabetes. 2023 Mar 29;8:e45241. doi: 10.2196/45241 (PMC10132000; doi:10.2196/45241)
Supplement: Multimedia Appendix 2 [file diabetes_v8i1e45241_app2.docx]

**Appendix 2**

Codebook based on the modified Theory of Planned Behavior.

- Knowledge and attitudes
  - Negative views
    - Clinical limitations
    - Psychosocial limitations
  - Positive views
    - Clinical benefits
    - Psychosocial benefits
- Network support
  - Challenges
  - Clinic resources
  - Non-clinic resources
  - Prescribing device
    - Device patient education
    - Device set-up
    - Monitoring and follow-up
    - Ordering device
- Perceived behavioral control
  - Clinician perceptions of patient self-efficacy
  - Clinician self-efficacy
- Use pattern
  - Predictors of discontinuation
  - Predictors of intermittent use
  - Predictors of persistent use and success
