# Supplementary material for: Clinician Experiences With Hybrid Closed Loop Insulin Delivery Systems in Veterans With Type 1 Diabetes: Qualitative Study
Source: JMIR Diabetes. 2023 Mar 29;8:e45241. doi: 10.2196/45241 (PMC10132000; doi:10.2196/45241)
Supplement: Multimedia Appendix 3 [file diabetes_v8i1e45241_app3.docx]

**Appendix 3**

Consolidated Criteria for Reporting Qualitative Research (COREQ): a 32-item checklist for interviews and focus groups.^15^

**Domain 1: Research Team and reflexivity**

Personal Characteristics

- 1. Interviewer/facilitator – Author HT conducted all interviews
  2. Credentials – Author’s credentials as listed in “Authors” section
  3. Occupation – Research team members included Endocrinologists, a pharmacist, pharmacy resident, and research assistants/specialists
  4. Gender – Research team included only females
  5. Experience and training – Author HT trained with a qualitative research specialist prior to conducting the research interviews

Relationship with participants

- 1. Relationship established – No relationship was established prior to study commencement with HT, the author that conducted the interviews
  2. Participant knowledge of interviewer – Study participants knew the interviewer/author, HT was a pharmacy resident at the Ann Arbor VA completing a research project
  3. Interviewer characteristics – No characteristics were reported

**Domain 2: Study design**

Theoretical framework

- 1. Methodological orientation and Theory – Using a descriptive approach, semi structured interviews were conducted then analyzed according to inductive thematic analysis

Participant selection

- 1. Sampling – Endocrinologists and Endocrinology fellows working within the University of Michigan and VA Ann Arbor Healthcare System
  2. Method of approach – Email
  3. Sample size – 13 participants approached, 11 total participants enrolled in the study
  4. Non-participation – No enrolled clinicians refused to participate or dropped out of the study

Setting

- 1. Setting of data collection - Interviews were conducted telephonically. Interviewer was located at the Ann Arbor VA.
  2. Presence of non-participants – No one else was present besides primary interview. Interviews were audio recorded to be analyzed by the other members of the research team.
  3. Description of sample – As described in Table 1. Participant Characteristics

Data Collection

- 1. Interview guide-Yes, interviews were conducted based on the pre-developed semi structed interview script in Supplement 1 of the Appendix
  2. Repeat Interviews – No interviews needed to be repeated
  3. Audio/visual recording – Yes, interviews were audio recorded using Audacity and Olympus DVR software
  4. Field notes – Yes brief notes were recorded after each interview
  5. Duration – Interviews lasted an average of 37 minutes
  6. Data saturation - Yes, study captured information from participants at our health system. Had we been at a different health system then we might be able to capture different information.
  7. Transcripts returned – No

**Domain 3: Analysis and findings**

Data analysis

- 1. Number of data coders – One, author HT
  2. Description of the coding tree – Yes, as described in Supplement 2 of the Appendix
  3. Derivation of themes – Themes were derived from interview data using thematic analysis techniques and in discussion with members of the larger research team
  4. Software – Nvivo12 software was utilized to manage the interview data
  5. Participant checking – No, participants did not provide feedback on the findings

Reporting

- 1. Quotations presented – Yes, participant quotes were presented and included to illustrate the themes/findings
  2. Data and findings consistent – Yes
  3. Clarity of major themes – Yes
  4. Clarity of minor themes – Yes
